# Millimeter-Wave Seed Priming Alters the Rice Root Proteomic Response to Salt Stress

**DOI:** 10.3390/biology15211921

**Published:** 2026-10-23

**Authors:** Setsuko Komatsu, Naozumi Katou, Takashi Furuya, Kouichi Kasahara, Hisateru Yamaguchi, Keisuke Hitachi, Masahiko Tani, Yuji Imura, Kunihiro Tsuchida

**Affiliations:** 1 Faculty of Environment and Information Sciences, Fukui University of Technology, Fukui 910-8505, Japan; 2 Development of Far-Infrared Region, University of Fukui, Fukui 910-8507, Japan; 3 Fukui Seed Co., Ltd., Sakai 919-0526, Japan; 4 Department of Management Nutrition, Aichi Gakusen University, Okazaki 444-8520, Japan; hyamaguchi@gakusen.ac.jp; 5 Center for Medical Science, Fujita Health University, Toyoake 470-1192, Japantsuchida@fujita-hu.ac.jp (K.T.)

**Keywords:** millimeter-wave irradiation, salt stress, seed priming, *Oryza sativa*, root proteome, antioxidant response

## Abstract

Salt stress is a major constraint on rice production because it inhibits plant growth and reduces yield. Although millimeter-wave (MMW) seed priming was reported to improve plant performance under environmental stress, the molecular mechanisms underlying its effects remain largely unknown. This study examined whether MMW seed priming improves early rice performance under salt stress and characterized associated changes in the root proteome. Rice seeds were irradiated with MMW, and morphological traits were evaluated under 150 mM NaCl as salt stress for 2 days. Based on morphological results, rice seeds were irradiated with 20 mW for 20 min. Root proteins were analyzed based on the growth responses. Among 6307 proteins analyzed with label-free proteomics, selected proteomic observations were further examined by immunoblotting, gene-expression analysis, and starch measurements. MMW irradiation improved seed germination and alleviated salt-induced inhibition of root growth. Proteomic analysis showed that proteins associated with carbohydrate metabolism and endoplasmic-reticulum function were markedly affected by MMW irradiation. Glutathione reductase and ascorbate peroxidase accumulated under salt stress and further increased following MMW irradiation. Calreticulin and calcium-dependent protein kinase were also increased in abundance by MMW irradiation under salt stress. In addition, soluble starch content, which declined under salt stress, was attenuated the salt-associated reduction by MMW irradiation. These findings extend molecular studies of MMW-mediated salt-stress responses to the rice root proteome and identify candidate proteins and biological processes associated with the improved salt-tolerant phenotype. The observed proteomic changes provide insight into potential mechanisms that may contribute to improved rice growth under saline conditions.

## Simple Summary

Rice production is severely affected by soil salinity worldwide. Although millimeter-wave seed priming has been reported to enhance plant performance under environmental stress, the molecular mechanisms responsible for this effect remain poorly understood. Proteomic analysis demonstrated that millimeter-wave priming reprogrammed the rice root proteome under salt stress. Improved root growth was associated with increased abundance of proteins associated with antioxidant defense by antioxidant scavenging systems, increased abundance of proteins associated with antioxidant defense, and regulation of changes in starch accumulation associated with altered carbohydrate-metabolism proteins. Together, these responses suggest that millimeter-wave priming was associated with coordinated changes in antioxidant-related, protein-homeostasis-related, and carbohydrate-metabolic processes. The results provide a molecular framework for the use of millimeter-wave priming as a potential nonchemical seed-treatment approach warranting further evaluation for saline agriculture.

## 1. Introduction

Rice is one of the most important staple crops worldwide, and its production is increasingly threatened by soil salinity, one of the most serious abiotic stresses limiting crop productivity [1,2]. Salt stress adversely affects seed germination, seedling establishment, root growth, and biomass accumulation, ultimately reducing grain yield [3,4]. The detrimental effects of salinity are primarily associated with ion toxicity, osmotic stress, and secondary oxidative stress caused by excessive accumulation of reactive oxygen species (ROS) [5,6,7,8,9]. To mitigate oxidative damage, plants activate antioxidant defense systems and other adaptive responses that contribute to stress tolerance [10]. Therefore, understanding the molecular mechanisms underlying salt-stress tolerance during early growth stages is important for improving rice performance under saline conditions.

Seed priming is a pre-sowing treatment widely used to improve seed germination, seedling establishment, and tolerance to environmental stresses. Various priming approaches, including hydropriming, osmopriming, biopriming, and physical priming, have been reported to enhance plant performance by inducing physiological and molecular changes before germination [11]. Among these approaches, millimeter-wave (MMW) irradiation has attracted increasing attention as a non-chemical physical seed-priming technology. On the other hand, MMWs are non-ionizing electromagnetic radiation whose interactions with biological materials depend on frequency, power density, exposure duration, and tissue water content [12]. The MMW frequency band ranges from 30 to 300 GHz, with wavelengths between 10 and 1 mm [13], and its application is growing rapidly. In brown rice, MMW irradiation promoted seed germination [14]. Early-stage MMW irradiation of wheat seeds improved not only the seed germination rate [15], but also plant growth and grain yield [16]. MMW irradiation of wheat seeds promotes the recovery of plant growth from salt stress by regulating ROS scavenging systems [17]. These observations raise the possibility that MMW seed treatment may improve rice performance under salinity.

Proteomic analysis is a valuable approach for investigating plant responses to abiotic stresses because proteins are the functional molecules directly involved in cellular regulation and stress adaptation. Previous studies have shown that salt tolerance in rice involves coordinated regulation of redox homeostasis, signaling networks, protein quality control, and metabolic processes [18,19]. Proteomic analyses have further demonstrated that salt stress induces extensive changes in proteins associated with these biological functions, highlighting the importance of protein-level characterization for understanding stress adaptation.

This study investigated whether MMW seed irradiation improves early rice performance under salt stress and characterized associated changes in the root proteome. Selected candidate responses were subsequently examined by immunoblotting and transcript analysis, and changes associated with carbohydrate metabolism were complemented by starch measurements.

## 2. Materials and Methods

### 2.1. Plant Material, MMW Irradiation, and Salt Treatment

The seeds of rice (*Oryza sativa* L. cultivar Koshihikari) were irradiated using a previous protocol [20]. Briefly, the frequency tuning range of the Signal Generator Extension Modules (SGX; Virginia Diodes, Charlottesville, VA, USA) was 75 to 110 GHz and the output power was 100 mW. The SGX was used in free-running mode at 110 GHz for the irradiation. The electromagnetic waves emitted from the SGX were passed through an isolator. After adjusting the output power using an attenuator, the electromagnetic wave was directed to free space through a horn antenna with an aperture angle of 17 degrees on each side. A Petri dish, which is 35 mm in diameter, containing 200 dry seeds was placed at a distance of 15 cm from the horn antenna so that the MMW irradiation area completely covered the entire dish. Seeds handled identically and positioned in the apparatus with the source disabled. MMW irradiation was performed under conditions where the exposure time was fixed at 20 min and the output power was set to 0, 10, 20, and 40 mW, or where the output power was fixed at 20 mW and the exposure time was set to 0, 10, 20, and 40 min. The average electromagnetic-wave intensities were 0.263, 0.525, and 1.5 mW/cm^2^ for output powers of 10, 20, and 40 mW, respectively. The irradiated electromagnetic wave has a Gaussian distribution with a maximum intensity of 1.20 times the average intensity at the center of the beam and a minimum intensity of 0.89 times the average intensity at the rim of the beam. Under the aforementioned irradiation conditions, the temperature rise of the rice was estimated to be well below 1 K. Accounting for heat dissipation, the temperature rise is approximately 0.13 °C.

**Figure 1:**
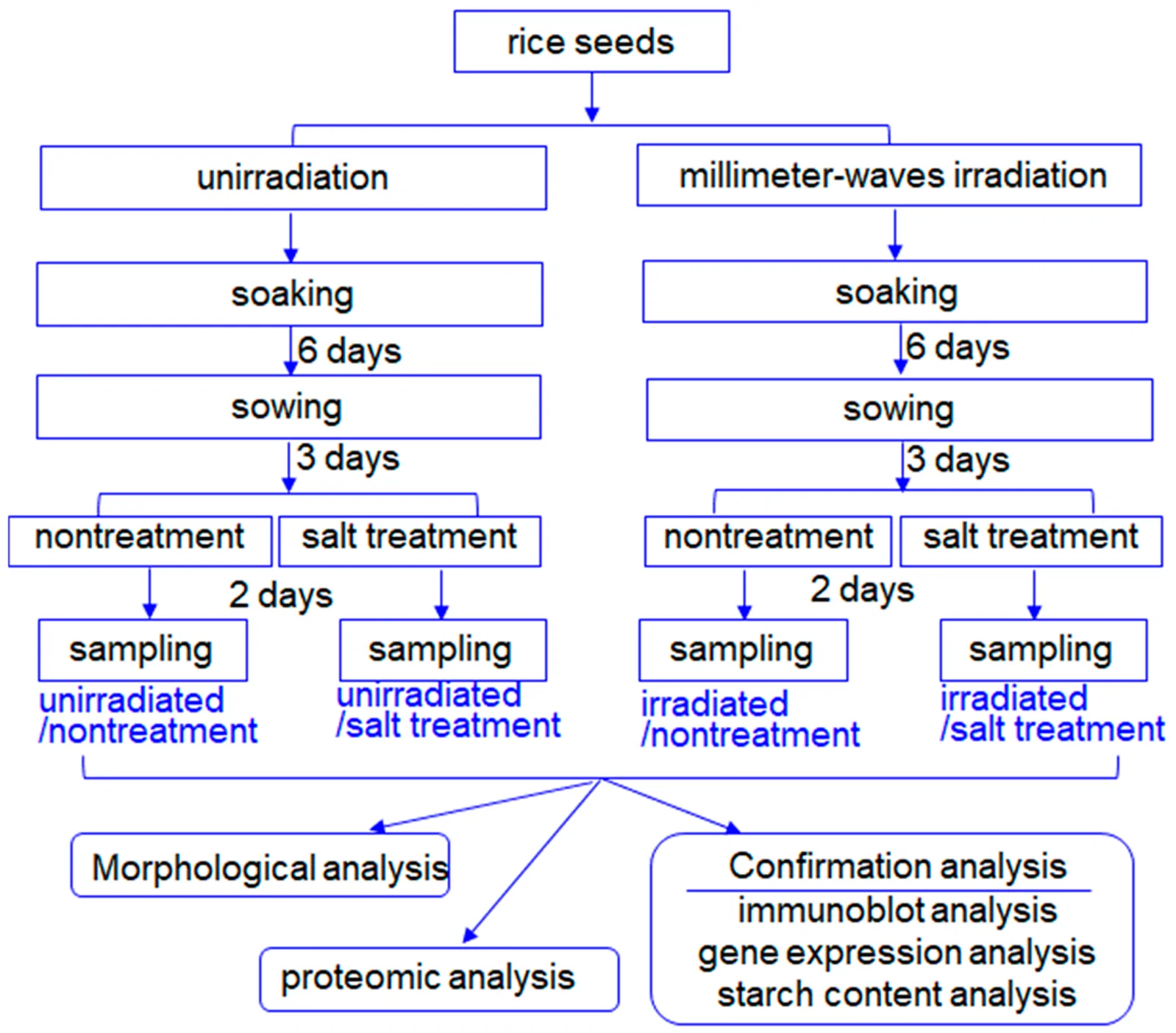
The experimental design for the investigation of the effect of MMW on rice under salt stress. Rice seeds were irradiated with MMW. After 6 days of soaking, rice was sowed. After 3 days of sowing, rice was treated with or without 150 mM NaCl (salt) for 2 days. Based on morphological analysis, proteomic analysis was conducted. For confirmation experiments, immunoblot, gene expression, and starch content analyses were used. All experiments were performed with more than 3 independent biological replicates. The samples were used in a fresh state, without being frozen.

After irradiation, the seeds were soaked for 6 days and sown in 400 mL of silica sand within seedling trays. A total of 20 seeds were sown evenly in each tray for each treatment. The rice was grown under white fluorescent light (160 µmol/m^2^·s^−1^; 16 h light period per day) at 25 °C and 60% humidity. Germination was evaluated using 20 seeds per biological replicate, and 3 independent experiments were conducted on different days (*n* = 60 seeds per treatment) for 6 days. The group receiving only water treatment served as the control. After 3 days of sowing, the rice was treated with 150 mM NaCl (salt) for 2 days (Figure 1). For measurements of shoot length, root length, shoot fresh weight, and root fresh weight, 16 seedlings with average growth were analyzed in each biological replicate. For other biological experiments, 16 seedlings with average growth were also analyzed in each biological replicate. Data were obtained from 3 independent experiments and are presented as mean ± SD.

### 2.2. Protein Extraction and Concentration Measurement

A portion (500 mg; fresh weight) of samples was ground in 500 µL of lysis buffer consisting of 50 mM Tris-HCl (pH 7.6), 100 mM NaCl, 1% Nonidet-P40, 0.1% SDS, and protease inhibitor (Nacalai Tesque, Kyoto, Japan) into a mortar and pestle on ice. The suspension was centrifuged at 16,000× *g* for 10 min at 4 °C, and the supernatant was centrifuged again under the same conditions. This supernatant was used as the crude protein extract. Proteins were reacted with XL-Bradford (Apro Science, Tokushima, Japan) and absorbance was measured at 595 nm. A standard curve for determining protein concentration was prepared using bovine serum albumin (BSA).

### 2.3. Proteomic Analysis

#### 2.3.1. Protein Enrichment, Reduction, Alkylation, and Digestion

Quantified proteins (100 µg) were adjusted to a final volume of 100 µL. Proteins were enriched, reduced, alkylated, and digested using previous methods [21]. Briefly, methanol (400 µL) was added to each sample followed by 100 µL of chloroform and 300 µL of water. After centrifugation at 16,000× *g* for 10 min, the upper and lower phases were discarded and 300 µL of methanol was added. After centrifugation at 16,000× *g* for 10 min, the pellet was collected and resuspended in 50 mM ammonium bicarbonate and reduced with 50 mM dithiothreitol at 56 °C for 30 min, followed by alkylation with 50 mM iodoacetamide at 37 °C for 30 min in the dark. These concentrations are final concentrations, and the final liquid volume is 100 µL. After this incubation, digestion was performed using trypsin (FUJIFILM Wako Pure Chemical, Osaka, Japan) at an enzyme-to-protein ratio of 1:100 at 37 °C for 18 h. The peptides were desalted using a MonoSpin C18 column (GL Sciences, Tokyo, Japan) and acidified with 1% trifluoroacetic acid. Samples were applied to liquid chromatography (LC)- mass spectrometry (MS)/MS.

#### 2.3.2. Protein Identification Using nanoLC-MS/MS

The conditions of LC (EASY-nLC 1000; Thermo Fisher Scientific, San Jose, CA, USA) and MS (Orbitrap Fusion ETD MS; Thermo Fisher Scientific) were described in the previous study [22]. All samples were analysed with the same LC and MS methods. Briefly, peptides equivalent to 1 µg (injection volume 1 µL) were loaded onto the LC system equipped with a trap column (Acclaim PepMap 100 C18 LC column, 3 µm, 75 µm ID × 20 mm; Thermo Fisher Scientific), equilibrated with 0.1% formic acid, and separated on the analytical column (EASY-Spray C18 LC column, 3 µm, 75 µm ID × 150 mm; Thermo Fisher Scientific) at a flow rate of 300 nL min^−1^ with a linear acetonitrile gradient (0–35%) in 0.1% formic acid over 120 min, followed by 35–95% over 5 min and 95% for 5 min (column wash; total method length 130 min). The equilibration volumes were 12 µL for the trap column and 4 µL for the analytical column. The eluted peptides were ionized with a spray voltage of 2 kV (ion transfer tube temperature: 275 °C). The peptide ions were detected using MS in the data-dependent acquisition mode (cycle time 3 s) with the installed Xcalibur software (version 4.7.69.37; Thermo Fisher Scientific). Full-scan mass spectra were acquired in the Orbitrap over 375–1500 *m*/*z* with a resolution of 120,000 (AGC target 4e5, maximum injection time 50 ms). Within each cycle, the most intense precursor ions were selected in order of decreasing intensity; precursor ions with charge states 2–7 and an intensity of at least 5000 were selected with monoisotopic precursor selection (peptide mode), isolated in the quadrupole (isolation window 1.6 *m*/*z*), and fragmented by collision-induced dissociation in the linear ion trap with a normalized collision energy of 35% (activation time 10 ms, activation Q 0.25). MS2 spectra were acquired in the linear ion trap at the Rapid scan rate (AGC target 2e3, maximum injection time 35 ms). Dynamic exclusion was applied for 60 s after one selection (±10 ppm, isotopes excluded). The twelve samples were acquired in blocks by group without randomization, with blank runs between samples; no pooled QC sample was used, and a BSA standard was measured before and after the sample series to check instrument performance.

#### 2.3.3. MS Data Analysis

The MS/MS searches were carried out using MASCOT (version 2.6.2; Matrix Science, London, UK) and SEQUEST HT search algorithms against the Protein Database (IRGSP1.0_protein_2024-07-12.fasta download from The Rice Annotation Project; https://rapdb.dna.affrc.go.jp/index.html, accessed on 9 September 2024) using Proteome Discoverer (version 2.4.1.15; Thermo Fisher Scientific). The workflow was described in the previous study [23]. Spectrum files RC, spectrum selector, MASCOT, SEQUEST HT search nodes, percolator, ptmRS, and Minora Feature Detector nodes were part of both algorithms. Carbamidomethylation of cysteine was set as a fixed modification. Methionine oxidation and protein N-terminal acetylation were set as variable modifications in both engines; Met-loss and Met-loss + acetylation were additionally allowed in SEQUEST HT. The precursor and fragment mass tolerances were 10 ppm and 0.6 Da, respectively. Trypsin was specified as the protease with up to 2 missed cleavages, and the peptide length was 6–144 residues. The FASTA file used was IRGSP-1.0_protein_2024-07-12.fasta (RAP-DB, version 2024-07-12; 42,761 entries). PSMs from MASCOT and SEQUEST HT were validated with Percolator using target-decoy searches. Target FDR thresholds of 0.01 (strict) and 0.05 (relaxed) were set for PSMs, peptides, and protein groups (Protein FDR Validator). Only peptides of high confidence were kept (peptide confidence filter: High), and lower-confidence PSMs were not kept; no filter on protein confidence was applied. The Thermo template workflows PWF_Tribrid_Precursor_Quan_and_LFQ_ITHCD_SequestHT_Mascot_Percolator_IMPptmRS (processing) and CWF_Comprehensive_EnhancedAnnotation_LFQ_and_Precursor_Quan_ContrastHigh (consensus) were used without modification. The protein table contained 5812 high-confidence groups (FDR ≤ 1%) and 495 medium-confidence groups (FDR 1–5%), and no low-confidence groups. The MASCOT and SEQUEST HT results were combined within the Proteome Discoverer workflow, with no changes to the settings and no additional reconciliation applied by us. Proteins were inferred by strict parsimony, and a minimum of one peptide sequence per protein was required, resulting in 6307 protein groups (6378 before parsimony; 1586 groups supported by a single peptide). No contaminant database was used; the search database contained rice proteins only.

#### 2.3.4. Differential Analysis of Proteins Using MS Data

Label-free quantification was performed with Proteome Discoverer using precursor-ions quantifier nodes. No additional sample-wise normalization was applied at this stage. Principal component analysis was also conducted in Proteome Discoverer. Downstream statistical analyses were performed using Perseus (version 1.6.15.0) [24]. Protein abundances were log_2_-transformed. Each group consisted of 3 biological replicates, and proteins with at least 3 valid values in at least one of the four groups were retained (5520 of 6307 protein groups). To reduce systematic global differences in signal intensity across samples, the median value of each sample was then subtracted (global median centering), assuming that the majority of proteins are not differentially expressed. Missing values in the retained proteins (1908 values in 717 proteins) were then imputed from a normal distribution (width = 0.3, down shift = 1.8), separately for each sample, on the assumption that they represented abundances too low to be quantified in that sample, because each retained protein had been quantified in all three replicates of at least one group. Statistical significance was assessed using Student’s *t*-test. Functional classification of differentially abundant proteins was performed using the Gene-Ontology database (http://www.geneontology.org/, accessed on 10 September 2024).

### 2.4. Immunoblot Analysis

SDS-sample buffer (BioRad, Hercules, CA, USA) consisting of 60 mM Tris-HCl (pH 6.8), 2% SDS, 10% glycerol, and 5% dithiothreitol was added to extracted proteins [25]. Quantified proteins (10 µg) were separated by electrophoresis on a 10% SDS-polyacrylamide gel and stained with Coomassie brilliant blue. On the other hand, for immunoblot, after electrophoresis, proteins were transferred onto a polyvinylidene difluoride (PVDF) membrane at 80 mA for 30 min using a semidry transfer blotter. The blotted PVDF membrane was blocked for 5 min in Bullet Blocking One regent (Nacalai Tesque), washed twice with TBS-T for 15 min, and cross-reacted with the primary antibodies (diluted 1:1000) for 30 min. As the primary antibodies, anti-superoxide dismutase (SOD) (Cat. 10269-1-AP; Proteintech, Rosemont, IL, USA), anti-ascorbate peroxidase (APX) [26], anti-peroxiredoxin (PRX) [27], anti-glutathione reductase (GR) (AS06 181; Agrisera, Vannas, Sweden), anti-calreticulin [28], anti-calnexin [29], calcium-dependent protein kinase (CDPK) [30], and heat shock protein 70 (Cat. SPC-302; StressMarq Biosciences, Victria, BC, Canada) were used. Anti-rabbit IgG conjugated with horseradish peroxidase (BioRad) was used as the secondary antibody (diluted 1:1000). After 30 min incubation, signals were detected using 3,3′,5,5′-tetramethylbenzidine solution (Nacarai Tesque) for 1 to 10 min. The integrated densities of bands were calculated using ImageJ software (version 1.53e, Java1.8.0 172, 64 bit; National Institutes of Health, Bethesda, MD, USA). All experiments were performed using three biological replicates for each sample.

### 2.5. RNA Extraction and Quantitative Real-Time Polymerase-Chain Reaction (qRT-PCR) Analysis

A portion (500 mg) of samples was snap-frozen in liquid nitrogen and ground into a pow der using a mortar and pestle. Total RNA was isolated with the RNeasy Plant Mini Kit (Qiagen, Venlo, The Netherlands) according to the protocol from the manufacturer. First-strand cDNA was synthesized from 1 μg of total RNA using the iSuperscript Reverse Transcription Supermix (Bio-Rad). Gene-specific primers were constructed with Primer3Plus software (https://www.bioinformatics.nl/cgi-bin/primer3plus/primer3plus.cgi/; accessed on 25 September 2026) [31] and used to amplify the 200–300 bp regions. A reaction mixture (20 μL) was used to perform qRT-PCR using SYBR Green Supermix (Invitrogen, Waltham, MA, USA). The PCR conditions included an initial step for 30 s at 95 °C, followed by 40 cycles of 10 s at 95 °C, and for 30 s at 60 °C. Gene expression was normalized using *18S rRNA* as an internal control. Relative expression was calculated according to the 2^−ΔΔct^ method.

### 2.6. Measurement of Starch Contents

A portion (10 mg; fresh) of samples was homogenized in phosphate-buffered saline with a mortar and pestle. Starch content was analyzed using a Starch Assay Kit (BioAssay Systems, Hayward, CA, USA). After washing away free glucose and low-molecular-weight oligosaccharides with 1 mL of 90% ethanol, the homogenate was heated at 60 °C for 5 min, with vortexing performed as appropriate during this time. After centrifugation at 10,000× *g* for 2 min, soluble starch in the pellet was extracted with 1 mL of water, incubated in a boiling water bath for 5 min, and then centrifuged at 10,000× *g* for 2 min. The supernatant was collected as soluble starch. After extracting the soluble starch, the water-insoluble precipitate was extracted with 0.2 mL of dimethyl sulfoxide and heated in a boiling water bath for 5 min. Each 10 μL sample was added to a working reagent by mixing 90 μL of assay buffer, 1 μL of enzyme A, 1 μL of enzyme B, and 1 μL of dye reagent. The mixture was incubated for 30 min at room temperature and the absorbance of the mixture was measured at 570 nm.

### 2.7. Statistical Analysis

The statistical significance of data between the 2 groups was analyzed using a Student’s *t*-test. A *p*-value of less than 0.05 was considered statistically significant.

## 3. Results

### 3.1. Seed Germination and Root Growth of Rice Following MMW Irradiation Under Salt Stress

**Figure 2:**
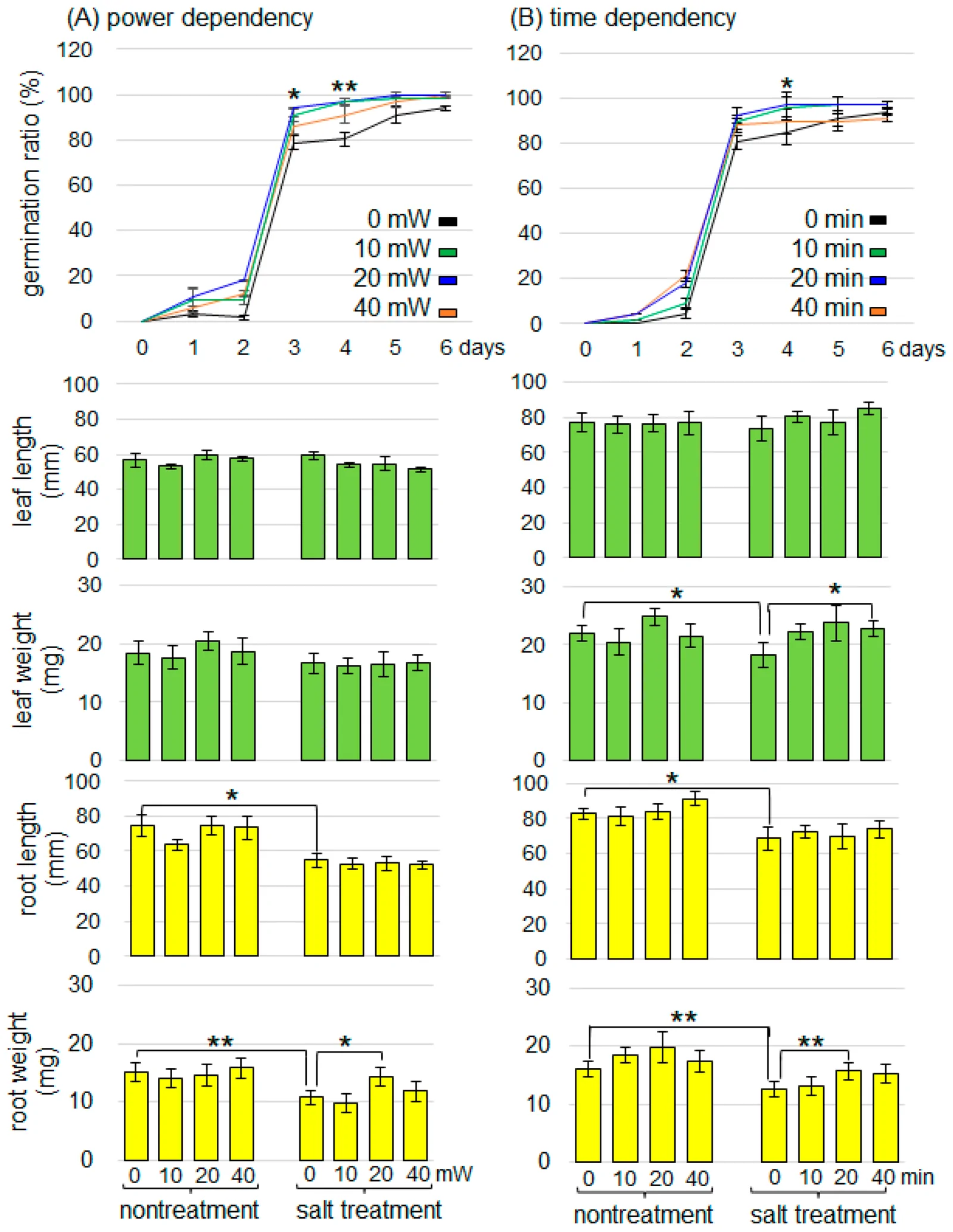
Morphological effects on MMW-irradiated rice treated with salt stress. Rice seeds irradiated with power dependency (**A**), which was 0, 10, 20, and 40 mW MMW, for time dependency (**B**), which was 0, 10, 20, and 40 min. After irradiation, seeds were soaked and measured germination ratio in every day. After 6 days of soaking, seeds were sowed. After 3 days of sowing, plant was treated without or with salt stress for 2 days and collected. As seedling collection, leaf (green column) and root (yellow column) were collected. As morphological parameters, leaf length, leaf-fresh weight, main root length, and total-root fresh weight were measured. The data are presented as mean ± SD from 3 independent biological replicates. Student’s *t*-test was used to compare values between 2 groups. Asterisks indicate significant changes (*, *p* < 0.05; **, *p* < 0.01).

To evaluate the effects of MMW irradiation on rice under salt stress, morphological parameters were analyzed. Rice seeds were irradiated with MMW at a fixed exposure time of 20 min and output powers of 0, 10, 20, and 40 mW (Figure 2A), or at a fixed output power of 20 mW and exposure times of 0, 10, 20, and 40 min (Figure 2B). Irradiated and unirradiated seeds were soaked for 6 days, and germination rates were recorded during that period. The point at which the shoot and root reached 5 mm or more in length was defined as germination. When the exposure time was fixed at 20 min, irradiation at 10 and 20 mW increased germination rates at 3 and 4 days after soaking compared with the unirradiated control. Similarly, when the output power was fixed at 20 mW, irradiation for 10 and 20 min increased germination rates at 4 days after soaking. In contrast, no improvement in germination was identified under higher irradiation intensity conditions (at 40 mW for 20 min, at 20 mW for 40 min) (Figure 2).

After 6 days of soaking, no significant differences in seedling growth parameters were observed between the non-irradiated and MMW-irradiated groups. The seeds were sown and grown for 3 days, followed by treatment with 150 mM NaCl for 2 days. In leaf, weight decreased under salt stress and increased with MMW irradiation at 20 mW for 40 min. Salt stress significantly reduced total root fresh weight and main root length in unirradiated rice seedlings (Figure 2). The reduction in total root fresh weight was attenuated the salt-induced reduction even if it was under salt stress when seeds irradiated with MMW irradiation at 20 mW for 20 min were sown (Figure 2). Based on these growth responses, an irradiation condition of 20 mW for 20 min was selected for subsequent experiments. Rice roots from these treatments were collected for proteomic analysis.

### 3.2. Identification and Functional Classification of Proteins in MMW-Irradiated Rice Under Salt Stress

**Figure 3:**
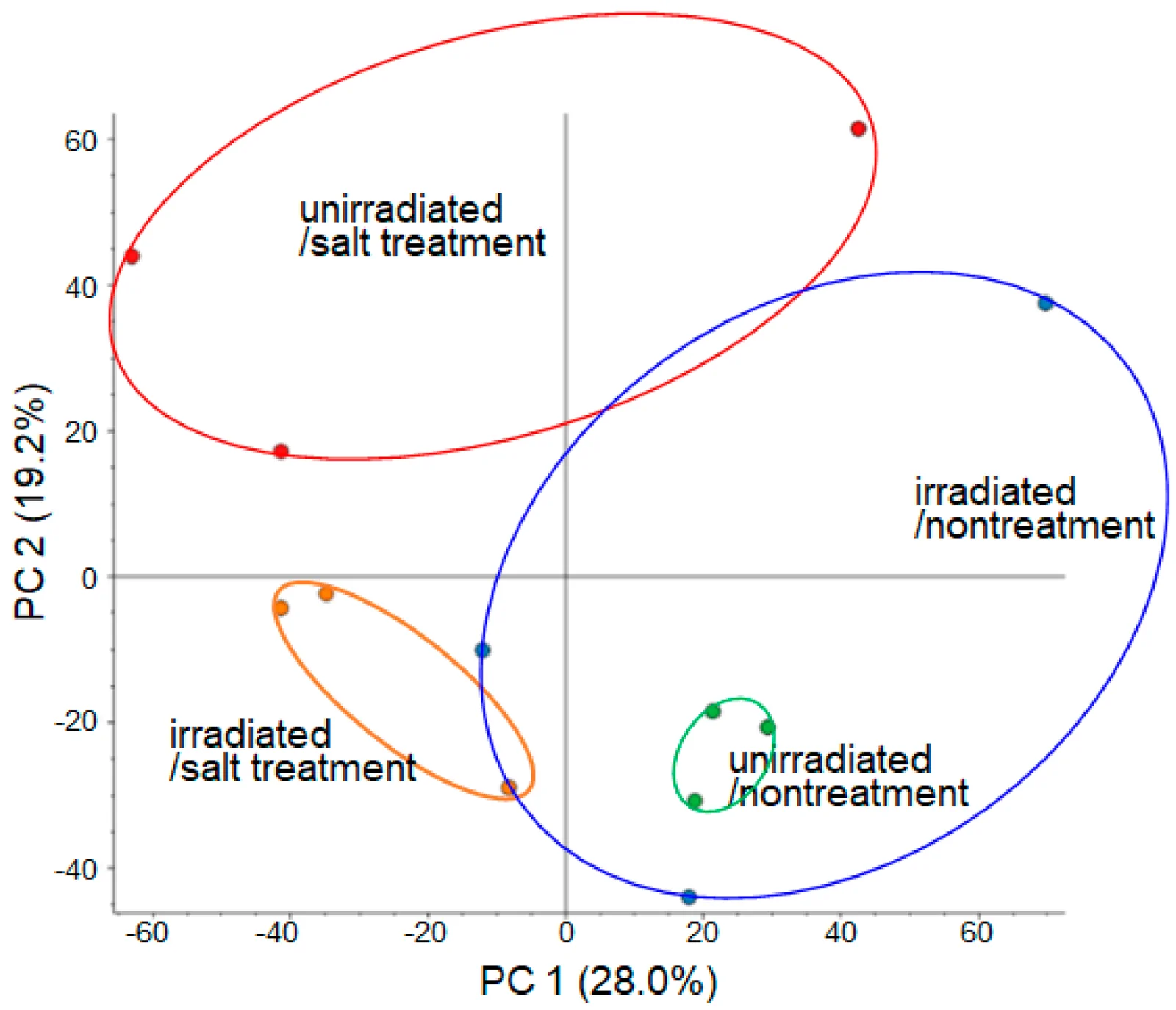
Overview of total proteomic data from 12 samples of root under salt stress of rice irradiated with MMW based on principal component analysis. Nine-day-old rice, whose seeds were irradiated with MMW, was exposed with or without salt stress for 2 days. Proteomic analysis was performed with 3 independent biological replicates for each treatment. Principal component analysis was performed with Proteome Discoverer.

**Figure 4:**
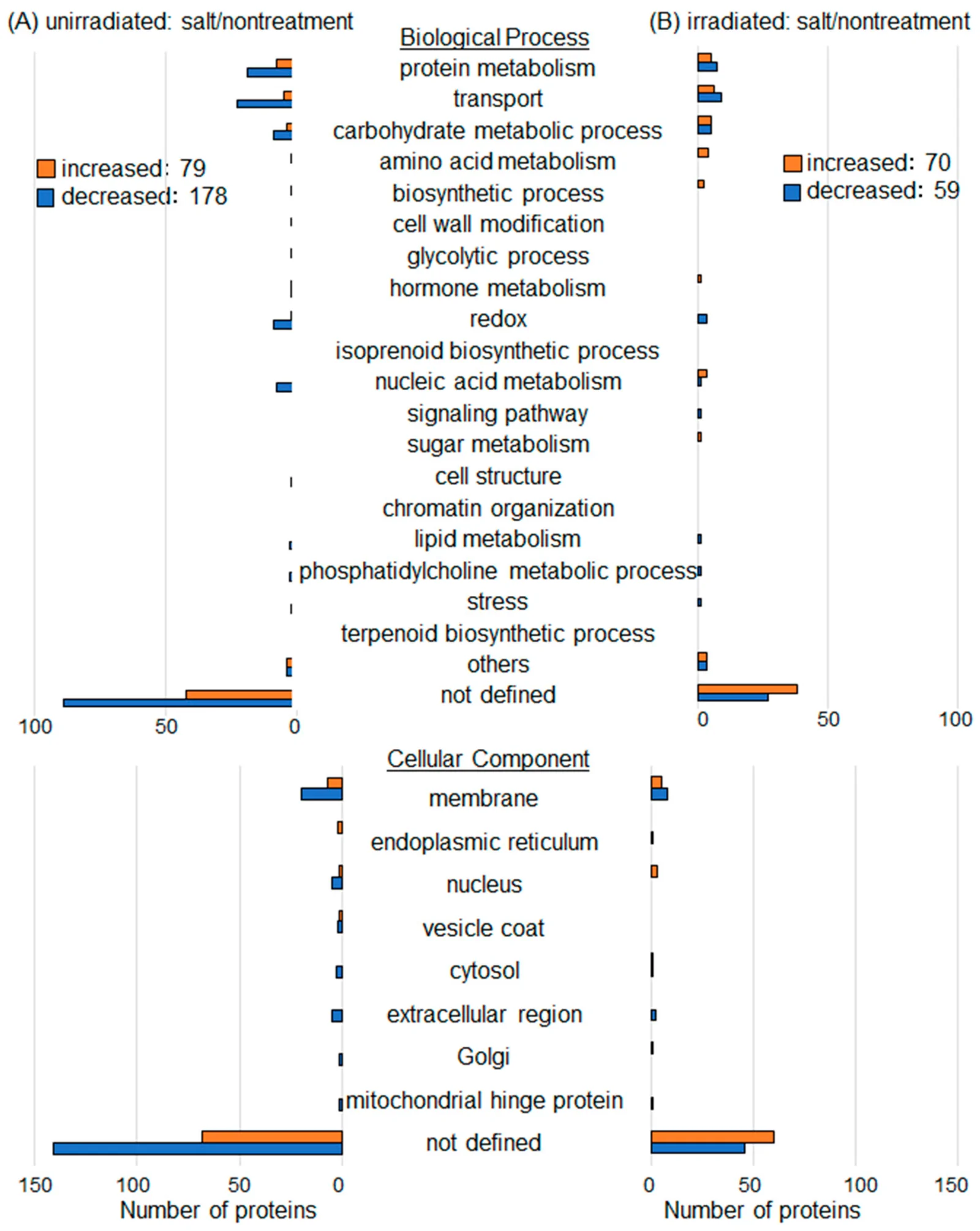
Functional categories of proteins with differential abundance in root of rice irradiated with MMW under salt stress. Four kinds of samples, which were unirradiated/nontreatment, unirradiated/salt treatment, irradiated/nontreatment, irradiated/salt treatment, were prepared. Proteins extracted from rice root were enriched, reduced, alkylated, and digested. After analysis by LC-MS/MS, the relative abundance of proteins from rice was compared as follows: salt/nontreatment without MMW irradiation (**A**) (Table S2) and salt/nontreatment with MMW irradiation (**B**) (Table S3). Functional categories of changed proteins were determined using Gene-Ontology analysis. Orange and blue columns show increased and decreased proteins, respectively.

To investigate the molecular mechanisms underlying the effects of MMW irradiation on rice growth under salt stress, a gel-free/label-free proteomic analysis was performed. Nine-day-old rice, whose seeds were irradiated with MMW, was exposed with or without salt stress for 2 days. Four treatment groups were analyzed: control (unirradiated, non-treated), salt stress (unirradiated, salt-treated), MMW irradiation (irradiated, non-treated), and MMW irradiation plus salt stress (irradiated, salt-treated) (Figure 3 and Figure 4). After treatment for 2 days, proteins extracted from rice whole roots were enriched, reduced, alkylated, and digested prior to nanoLC-MS/MS analysis. A total of 6307 proteins were identified, and their relative abundances were compared among the 4 treatment groups. Principal component analysis of the proteomic data from the 12 samples revealed distinct protein accumulation patterns among the four treatments (Figure 3). The separation of the salt-stressed samples from the control samples indicated that salt stress markedly altered the rice root proteome, whereas MMW irradiation induced additional changes in protein accumulation patterns under both normal and salt-stress conditions.

A total of 257 proteins showed differential accumulation (*p* < 0.05, fold change >1.5 or <0.67) in salt-stressed roots compared with the control (Table S2). Among these proteins, 79 increased and 178 decreased in abundance under salt stress (Figure 4A and Table S2). In addition, 129 proteins were differentially accumulated (*p* < 0.05, fold change >1.5 or <0.67) in MMW-irradiated roots under salt stress compared with MMW irradiation alone (Table S3). Of these proteins, 70 increased and 59 decreased in abundance (Figure 4B and Table S3). Gene Ontology analysis was performed to classify the differentially accumulated proteins (Figure 4). Proteins showing opposite accumulation patterns between the salt/control and irradiation + salt/irradiation comparisons were mainly associated with carbohydrate metabolism and nucleic acid metabolism in the biological process category, and with the endoplasmic reticulum and nucleus in the cellular component category (Figure 4).

### 3.3. Immunoblot Analysis of Proteins Related to ROS Scavenging and Ca^2+^ Signaling in MMW-Irradiated Rice Under Salt Stress

**Figure 5:**
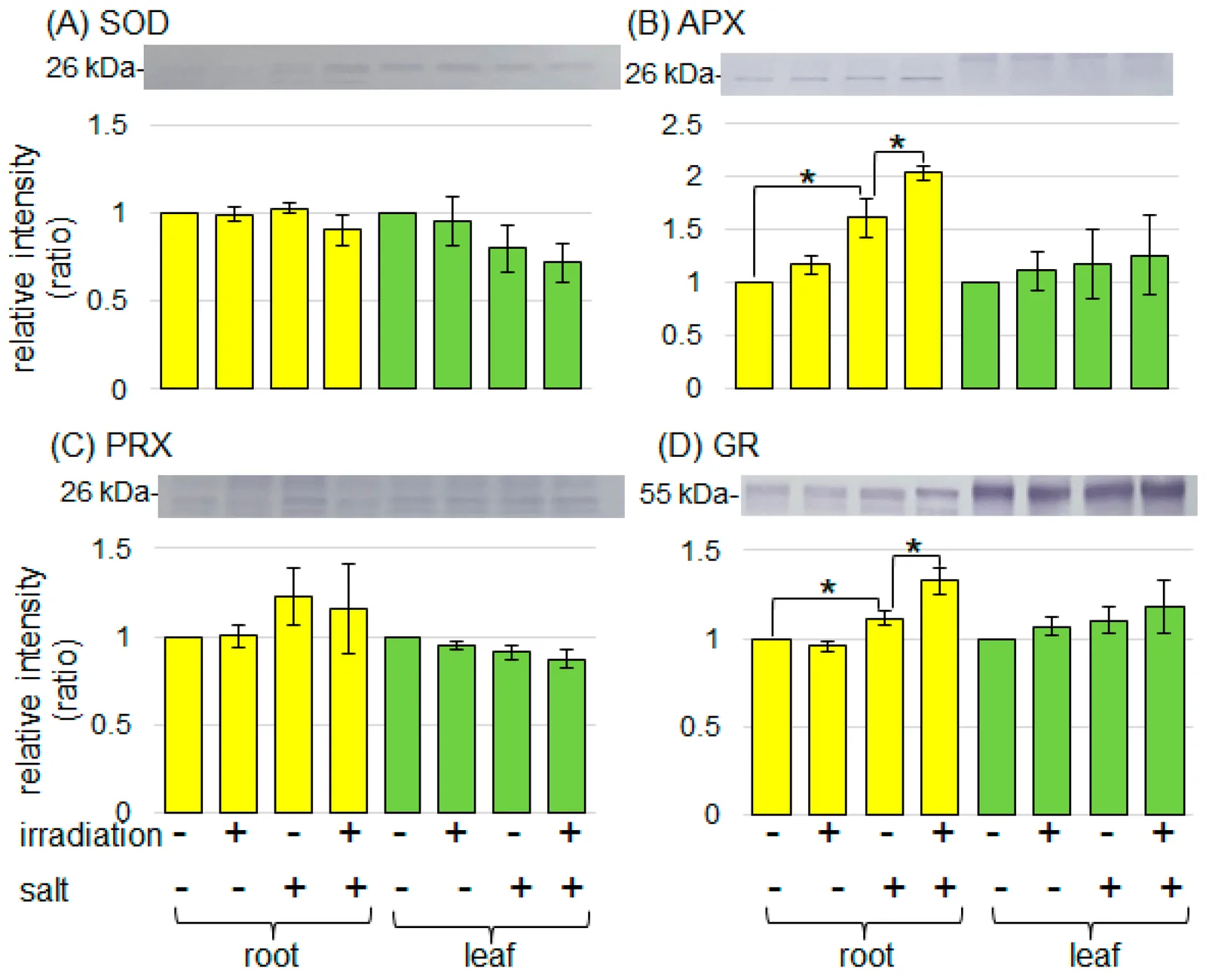
Immunoblot analysis of the ROS-related proteins involved in rice irradiated with MMW under salt stress. Proteins extracted from rice root and leaf were separated on SDS-polyacrylamide gel by electrophoresis and transferred onto PVDF membranes. The PVDF membranes were cross-reacted with anti-SOD (**A**), APX (**B**), PRX (**C**), and GR (**D**) antibodies. A staining pattern with Coomassie-brilliant blue was used as a loading control (Figure S1). The integrated densities of the bands were calculated using ImageJ software. The data are presented as mean ± SD from 3 independent biological replicates (Figures S2–S5). Student’s *t*-test was used to compare values between 2 groups. Asterisks indicate significant changes (*, *p* < 0.05).

**Figure 6:**
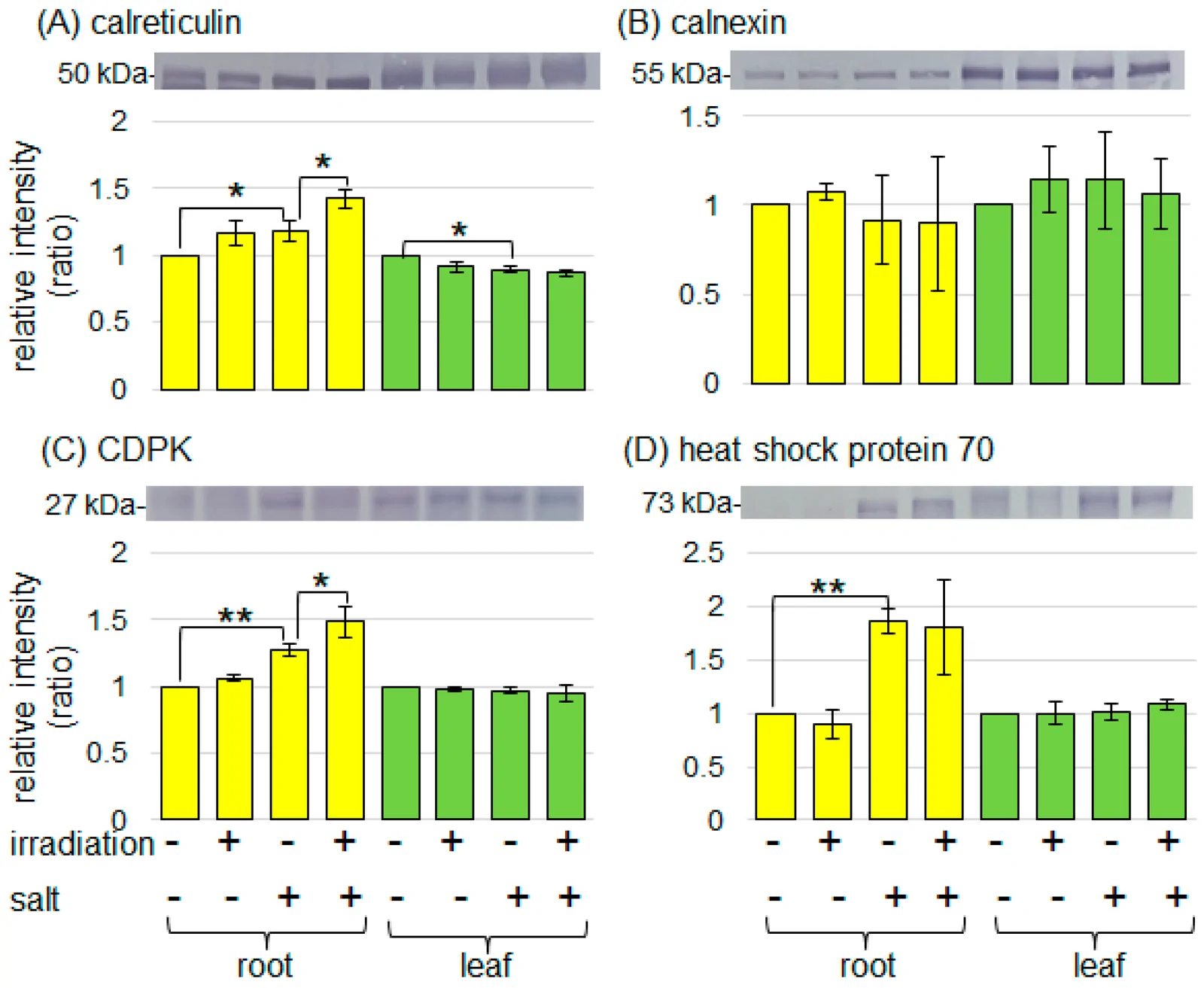
Immunoblot analysis of the calcium-related proteins involved in rice irradiated with MMW under salt stress. Proteins extracted from rice root and leaf were separated on SDS-polyacrylamide gel by electrophoresis and transferred onto PVDF membranes. The PVDF membranes were cross-reacted with anti-calreticulin (**A**), calnexin (**B**), CDPK (**C**), and heat shock protein 70 (**D**) antibodies. A staining pattern with Coomassie-brilliant blue was used as a loading control (Figure S1). The integrated densities of the bands were calculated using ImageJ software. The data are presented as mean ± SD from 3 independent biological replicates (Figures S6–S9). Student’s *t*-test was used to compare values between 2 groups. Asterisks indicate significant changes (*, *p* < 0.05; **, *p* < 0.01).

To further characterize the protein changes induced by salt stress and MMW irradiation, immunoblot analysis was performed using antibodies against proteins involved in ROS scavenging (Figure 5) and calcium signaling (Figure 6). Proteins extracted from rice roots and leaves were separated by SDS-polyacrylamide gel electrophoresis, and Coomassie brilliant blue staining was used as a loading control (Figure S1). Band intensities from 3 biological replicates (Figures S2–S9) were quantified using ImageJ software, and ratios were calculated by comparing the resulting integrated densities to the control (Figure 5 and Figure 6).

For the analysis of ROS-scavenging proteins, SOD (Figure 5A), APX (Figure 5B), PRX (Figure 5C), and GR (Figure 5D) were examined (Figures S2–S5). Cytosolic APX and GR levels in roots increased under salt stress and further increased following MMW irradiation under salt-stress conditions (Figure 5B,D). In contrast, no significant changes were identified in the levels of SOD or PRX (Figure 5A,C). In the leaves, ROS-scavenging proteins did not change. For the analysis of Ca^2+^ signaling- and protein-folding-related proteins, calreticulin (Figure 6A), calnexin (Figure 6B), CDPK (Figure 6C), and heat shock protein 70 (Figure 6D) were examined (Figures S6–S9). Calreticulin and CDPK accumulated in roots under salt stress and showed further increases in response to MMW irradiation under salt-stress conditions (Figure 6A,C). In contrast, calnexin levels did not change significantly, whereas heat shock protein 70 accumulated under salt stress but was not further affected by MMW irradiation (Figure 6B,D).

### 3.4. qRT-PCR Analysis of Genes Encoding Proteins Identified by Proteomic Analysis in MMW-Irradiated Rice Under Salt Stress

**Figure 7:**
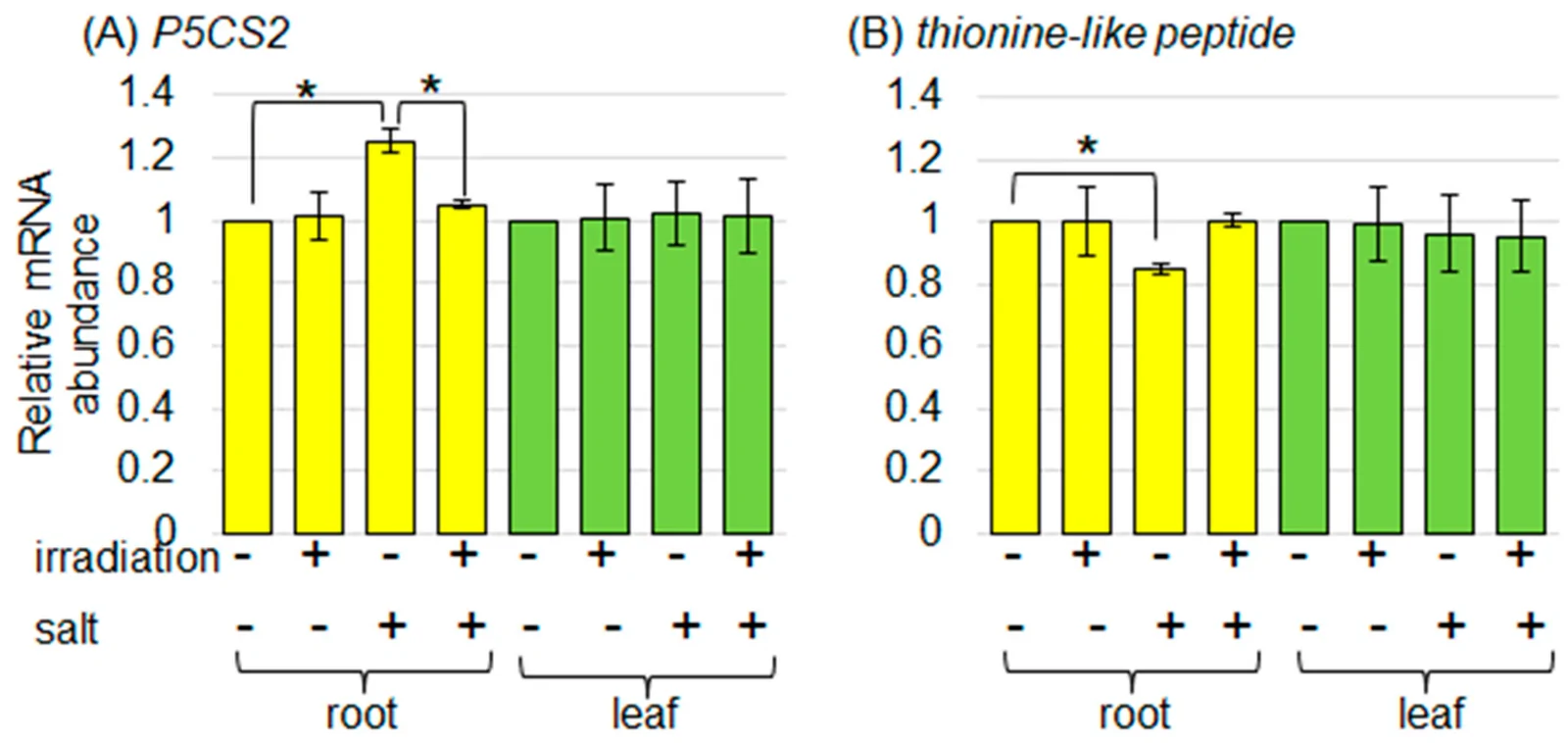
qRT-PCR analysis of genes encoding proteins identified by proteomic analysis in rice irradiated with MMW under salt stress. After extraction of total RNA from root and leaf, gene expression ratio of *P5CS2* (**A**) and *thionine-like peptide* (**B**) was analyzed by qRT-PCR. *18S rRNA* was used as an internal control. Data are shown as the means ± SD from 3 independent biological replicates. Student’s *t*-test was used to compare values between 2 groups. Asterisks indicate significant changes (*, *p* < 0.05).

To further examine the molecular responses associated with the proteins identified by proteomic analysis, qRT-PCR analysis was performed for selected protein-encoding genes (Figure 7). Gene-specific oligonucleotide primers were used to amplify transcripts from total RNA isolated from rice roots and leaves. The expression level of *18S rRNA*, used as an internal control, remained unchanged among the treatments. The expression levels of genes encoding proteins, which are P5CS2 (Figure 7A) and thionine-like peptide (Figure 7B), were analyzed in rice subjected to salt stress with or without MMW irradiation.

Among the analyzed genes, *P5CS2* expression was significantly upregulated under salt stress. However, its expression level was downregulated following MMW irradiation under salt-stress conditions and returned toward the control level (Figure 7A). In contrast, *thionine-like peptide* expression was downregulated by salt stress and was not significantly affected by MMW irradiation (Figure 7B).

### 3.5. Starch Contents in MMW-Irradiated Rice Under Salt Stress

**Figure 8:**
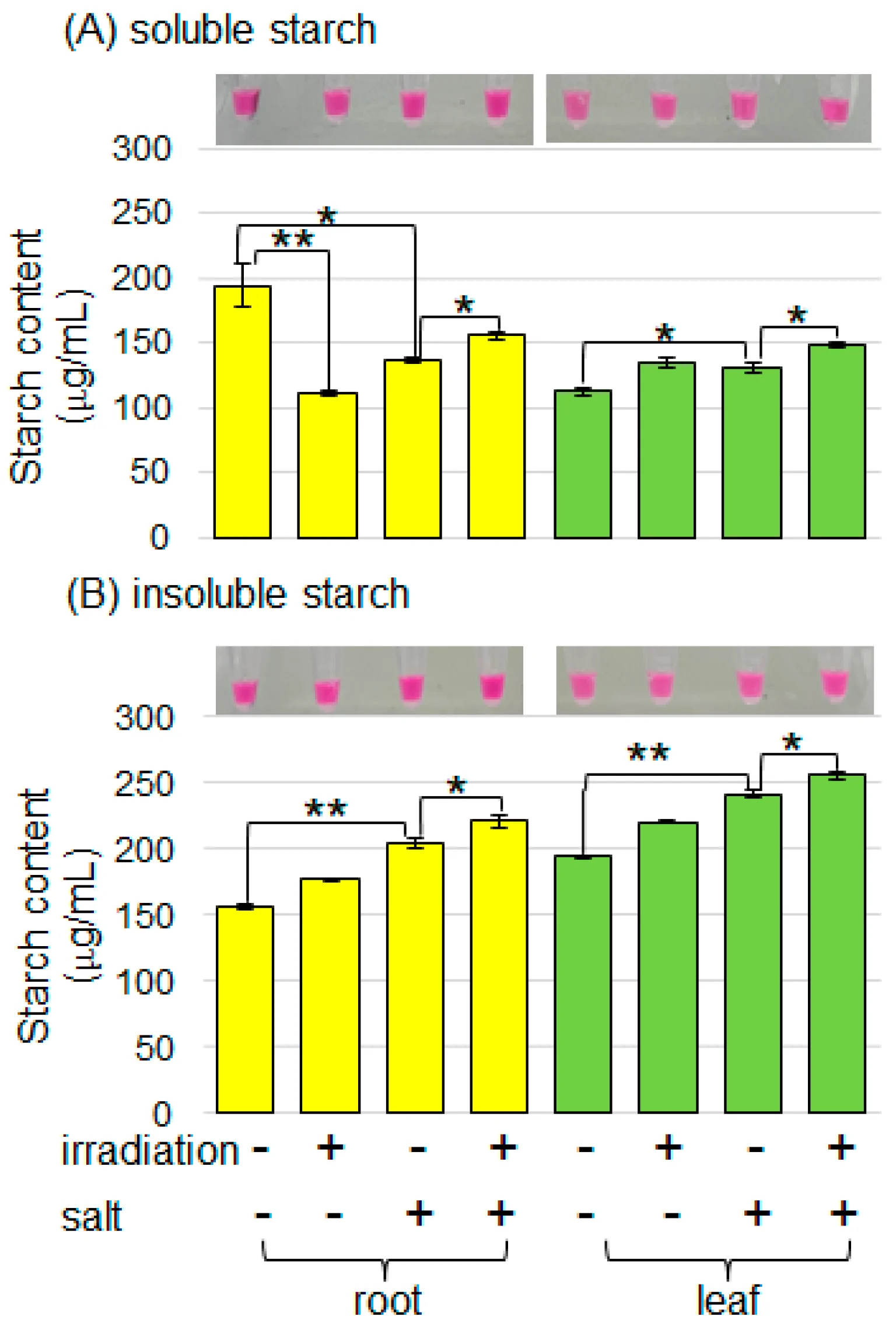
Contents of starch in rice irradiated with MMW under salt stress. After extraction of metabolites, the contents of soluble and insoluble starches were analyzed using a Starch Assay Kit. Data are shown as the means ± SD from 3 independent biological replicates. Student’s *t*-test was used to compare values between 2 groups. Asterisks indicate significant changes (*, *p* < 0.05; **, *p* < 0.01).

Because proteins associated with carbohydrate metabolism showed opposite accumulation patterns in rice roots under salt stress with and without MMW irradiation (Figure 4), starch contents were analyzed (Figure 8). In roots, soluble starch content decreased under salt stress but increased following MMW irradiation under salt-stress conditions, reaching levels comparable to those of the control (Figure 8A). In contrast, insoluble starch content increased under salt stress and showed a further increase in response to MMW irradiation (Figure 8B). In leaves, both soluble and insoluble starch contents increased under salt stress and were further elevated by MMW irradiation.

## 4. Discussion

### 4.1. MMW Irradiation of Rice Seeds Promotes Germination and Mitigates Salt Stress-Induced Growth Reduction

Salt stress is a major environmental constraint, which impairs seed germination, early seedling establishment, root development, and ultimately grain yield in rice [3]. Because successful germination and early root elongation are critical factors determining rice productivity [4], approaches, which improve plant performance during these developmental stages, are attracting significant interest from an agricultural perspective. The present study investigates whether MMW-irradiated seeds could improve rice germination and enhance tolerance to salt stress.

Previous studies demonstrated that electromagnetic-wave treatments can influence plant growth in a species- and dose-dependent manner. In wheat, MMW irradiation promoted seed germination [16], plant growth, and grain yield [15]. While similar growth-promoting effects are detected in certain plant species exposed to electromagnetic fields, excessive irradiation intensity or prolonged exposure can inhibit growth and germination [32,33,34]. Consistent with these findings, the present study demonstrated that MMW irradiation at 20 mW for 20 min promoted rice seed germination, whereas no further effects were detected under higher irradiation intensity conditions (Figure 2). These findings suggest that the effects of MMW irradiation on germination may depend on irradiation conditions. Although the underlying mechanism remains unclear, the observed response is consistent with the possibility that specific irradiation conditions are more effective than others in promoting germination.

Beyond its effects on germination, MMW irradiation enhanced plant tolerance to environmental stresses. Improved recovery from flooding stress was associated with the regulation of glycolysis, ROS scavenging, photosynthesis, and hormone-related pathways in soybean [35], chickpea [36], and wheat [37]. More recently, MMW-irradiated seeds improve wheat growth under salt stress by enhancing ROS scavenging capacity [17]. In agreement with these findings, MMW irradiation alleviated the reduction in rice root fresh weight caused by salt stress (Figure 2). Importantly, whereas previous studies of MMW-mediated salt-stress recovery primarily focused on physiological responses in wheat, the present study extends these observations by characterizing proteome-wide changes in rice roots under salt-stress conditions. This approach enabled the identification of candidate proteins and biological processes associated with the response to MMW seed priming. The present results indicate that MMW seed priming was associated with improved root growth under salt-stress conditions and with changes in the abundance of proteins involved in multiple biological processes. These proteins represent candidate factors associated with the observed phenotype and provide a basis for further investigation of molecular responses to MMW treatment under salinity.

### 4.2. MMW Priming Is Associated with Increased Abundance of Ascorbic Acid (AsA)–Glutathione (GSH)-Cycle Components Under Salt Stress

Salt stress disrupts cellular redox homeostasis and promotes excessive accumulation of ROS, leading to oxidative damage and growth inhibition. To limit ROS-induced injury, plants employ both enzymatic and non-enzymatic antioxidant systems. Among these defense mechanisms, the AsA-GSH cycle plays a central role in detoxifying hydrogen peroxide and maintaining cellular redox balance [38,39]. Key components of this cycle include APX, which reduces hydrogen peroxide using AsA as an electron donor, and GR, which regenerates reduced GSH, required for continuous antioxidant activity [40]. These reports suggest that the AsA-GSH cycle is key to the salt stress response.

Previous studies demonstrated that antioxidant enzymes are activated in response to salinity to counteract oxidative stress. Exogenous application of GSH enhances endogenous antioxidant capacity by increasing the activities of SOD, APX, and GR [6,41]. In rice roots, GR also accumulated following salt exposure [42]. Consistent with these observations, in this study, APX and GR accumulated under salt stress and increased further following MMW irradiation; in contrast, SOD and PRX did not exhibit significant changes (Figure 5). Interestingly, the response was selective because APX and GR increased following MMW treatment, whereas SOD and PRX showed no significant changes. This pattern suggests that MMW priming may be associated with specific antioxidant-related proteins rather than a generalized activation of the antioxidant network.

The increased abundance of APX and GR proteins following MMW irradiation suggests an association between MMW treatment and components of the AsA–GSH cycle. However, because enzyme activities, ROS levels, hydrogen peroxide content, and redox metabolites were not determined in the present study, the functional consequences of these protein changes cannot be directly assessed. Because oxidative stress is a major component of salt-induced cellular damage, the observed increases in APX and GR abundance may be associated with the improved root growth observed in MMW-irradiated rice under saline conditions. However, a direct relationship between these protein changes and stress mitigation remains to be verified. These findings identify APX and GR as candidate proteins associated with the response of rice roots to MMW priming under salt stress. Further studies involving direct measurements of ROS, hydrogen peroxide, antioxidant enzyme activities, and redox metabolites will be required to clarify their functional significance. A limitation of the present study is that ROS accumulation, hydrogen peroxide content, antioxidant enzyme activities, and redox metabolites were not measured. Therefore, the physiological significance of the observed protein changes requires further investigation.

### 4.3. MMW Priming Alters Proteins Associated with Ca^2+^ Signaling and ER Homeostasis Under Salt Stress

Calcium (Ca^2+^) serves as one of the earliest signaling molecules activated in response to salinity [43,44]. Salt stress rapidly induces transient increases in cytosolic Ca^2+^ concentrations, which initiate downstream signaling cascades involved in stress adaptation [45,46]. These Ca^2+^ signatures are decoded by Ca^2+^-binding proteins and protein kinases, which regulate ion transport, cellular homeostasis, and stress-responsive gene expression. Proteomic studies identified calreticulin and calmodulin as important components of the salt-stress response in rice [47]. The regulation of Ca^2+^-dependent events is considered to be one of the factors conferring salt stress tolerance in rice.

Calreticulin is an endoplasmic reticulum (ER)-resident molecular chaperone, which binds Ca^2+^ and contributes to both Ca^2+^ storage and protein folding [48,49]. In addition to maintaining Ca^2+^ homeostasis, calreticulin participates in ER quality-control systems, which preserve protein functionality under environmental stress [50]. Consistent with these roles, proteomic and immunoblot analyses identified that calreticulin accumulation increased under salt stress and was further enhanced by MMW irradiation (Figure 6). The Gene-Ontology analysis identified several proteins associated with endoplasmic-reticulum-related categories (Figure 4). Although these observations suggest a possible involvement of ER-associated processes in the response to MMW treatment, further studies will be required to determine their functional significance.

CDPK functions as a key mediator of Ca^2+^ signaling and associates with enhanced salt tolerance through the regulation of ROS balance and cellular protection mechanisms [51]. In the present study, CDPK levels increased under salt stress and accumulated further following MMW irradiation (Figure 6). Because CDPKs are known to participate in Ca^2+^-dependent stress responses and may interact with ROS-related pathways [51], the increased abundance of CDPK observed following MMW treatment suggests a possible association between MMW priming and proteins involved in stress signaling. However, because Ca^2+^ dynamics, CDPK activity, and downstream signaling events were not measured, the functional significance of this accumulation remains unclear. Interestingly, the response to MMW treatment was selective rather than uniform. Calreticulin accumulated following MMW treatment, whereas calnexin did not exhibit a comparable response. Similarly, HSP70 increased under salt stress but showed no additional accumulation following MMW irradiation. These observations suggest that MMW treatment may be associated with specific proteins involved in stress adaptation rather than a general increase in all ER-related or stress-responsive proteins. Taken together, the observed accumulation of calreticulin and CDPK suggests that MMW priming is associated with changes in proteins involved in Ca^2+^-related signaling and ER-associated functions under salt-stress conditions. These proteins represent candidate components of the molecular response to MMW treatment. However, because direct measurements of Ca^2+^ signaling, kinase activity, ER stress, and protein-folding status were not performed, their functional roles in MMW-mediated stress adaptation remain to be clarified.

### 4.4. MMW Priming Attenuates Salt-Induced P5CS2 Expression

Under salt stress, plants primarily regulate their osmotic potential through the accumulation of inorganic ions and the synthesis of compatible solutes [52]. However, excessive accumulation of inorganic ions can eventually reach toxic levels and cause secondary damage to plant cells [53]. Among compatible solutes, proline is one of the most extensively accumulated metabolites in plants subjected to abiotic stress. In addition to its role in osmotic adjustment, proline stabilizes proteins and membranes and contributes to the maintenance of cellular redox balance [54]. Proline is synthesized through the glutamate and ornithine pathways [55]. In the glutamate pathway, P5CS catalyzes the rate-limiting step by converting glutamate into glutamate-γ-semialdehyde, which is subsequently converted to pyrroline-5-carboxylate [56]. Because P5CS is a key regulatory enzyme in proline biosynthesis, enhanced P5CS activity generally promotes proline accumulation and improves osmotic adjustment under environmental stress conditions [57]. Therefore, *P5CS* expression is often used as a molecular indicator of stress-induced proline accumulation and closely associates with enhanced tolerance to salinity in a wide range of plant species.

Previous studies demonstrated a close relationship between *P5CS* expression, proline accumulation, and salt tolerance. Overexpression of the rice *P5CS* gene increased proline accumulation by 10- to 18-fold and significantly enhanced salt tolerance and root biomass [58]. Similar observations were reported in wheat [59], sugarcane [60], and maize [61], where elevated *P5CS* expression was associated with improved tolerance to salinity. Consistent with these reports, P5CS2 expression was significantly upregulated under salt-stress conditions in the present study. However, P5CS2 expression decreased toward the control level following MMW irradiation (Figure 7). These observations indicate that MMW treatment was associated with reduced accumulation of P5CS2 transcripts under salt stress. The biological significance of this response remains unclear because proline content, P5CS protein abundance, and P5CS activity were not determined in the present study.

However, because neither proline content nor P5CS protein abundance was determined in this study, the contribution of proline metabolism to the observed response cannot be excluded. Several alternative explanations may account for the reduced P5CS2 expression observed following MMW treatment. For example, MMW irradiation may reduce the intensity of stress perceived by rice roots, resulting in lower induction of stress-responsive genes. Alternatively, MMW treatment may affect the transcriptional regulation of P5CS2, induce compensatory metabolic responses, or alter proline turnover through pathways not examined in the present study. In addition, other components of proline metabolism, including P5CS1 and proline degradation pathways, were not analyzed. Therefore, the present data do not allow conclusions regarding the overall contribution of proline metabolism to MMW-mediated responses under salt-stress conditions. Direct measurements of proline accumulation together with analyses of P5CS protein abundance and enzyme activity will be required to clarify the functional significance of the observed changes in P5CS2 transcript levels.

### 4.5. MMW Priming Is Associated with Altered Starch Accumulation Under Salt Stress

Under salt stress, carbohydrate metabolism plays a central role in maintaining plant growth and supporting stress adaptation [62]. Carbohydrates contribute to plant defense mechanisms under abiotic stress by providing both metabolic energy and osmotic protection [63]. Previous studies indicated that soluble starch content progressively decreases during prolonged salt stress, reaching its lowest level after extended exposure [64]. In rice, the accumulation of soluble starch and soluble sugars contributes not only to osmotic adjustment but also to the maintenance of carbon and energy reserves, thereby supporting survival and productivity under saline conditions [65]. In contrast, insoluble starch functions as an osmotically inert carbon reserve that can be mobilized to sustain metabolism during environmental stress [66,67]. In the present study, starch was analyzed as soluble and insoluble fractions. The soluble starch fraction represents more readily extractable starch components, whereas the insoluble fraction represents less soluble starch reserves remaining in plant tissues after extraction. Because both fractions reflect starch abundance rather than metabolic activity, their physiological significance should be interpreted with caution. Therefore, changes in starch accumulation may represent one component of the plant response to saline conditions.

In the present study, proteins associated with carbohydrate metabolism exhibited altered accumulation patterns in response to salt stress and MMW treatment (Figure 4). In addition, soluble and insoluble starch contents differed among treatments, suggesting that MMW priming was associated with changes in starch accumulation under saline conditions. Consistent with these proteomic changes, soluble starch content decreased under salt stress but recovered to levels comparable to those of the control following MMW irradiation. In addition, insoluble starch content increased under salt stress and accumulated further in rice root and leaf in response to MMW treatment (Figure 8). The recovery of soluble starch and the increased accumulation of insoluble starch observed following MMW treatment indicate changes in starch abundance under saline conditions. However, because starch synthesis, degradation, carbon allocation, and energy status were not directly measured, the physiological significance of these changes remains unclear. Several explanations are possible, including altered starch synthesis, reduced starch mobilization, developmental effects associated with improved seedling growth, or changes in source–sink relationships. Together, these findings indicate that MMW priming was associated with altered starch accumulation under salt-stress conditions. The observed changes in starch content, together with alterations in carbohydrate-related proteins, identify carbohydrate metabolism as a candidate biological process associated with the response of rice plants to MMW treatment. Further studies will be required to determine the functional significance of these changes and their relationship to stress adaptation.

## 5. Conclusions

Although the physiological and proteomic responses induced by MMW irradiation were clearly observed in the present study, the primary mechanisms by which MMW signals are perceived and transduced in plant cells remain unclear. Because millimeter waves exhibit limited penetration into biological tissues and their interaction with intracellular aqueous environments is not fully understood, the initial targets of MMW irradiation remain a subject of debate. Previous studies have suggested that MMW exposure may influence membrane-associated processes, ion transport, and downstream signaling pathways. In addition, modifications in the organization and dynamics of water molecules surrounding biological macromolecules have been proposed as potential mechanisms. However, these hypotheses remain speculative and have not been conclusively demonstrated in plants. Further studies will be required to elucidate the biophysical basis of MMW perception and the signaling processes leading to stress adaptation.

This study provides proteomic insights into the response of rice roots to MMW seed priming under salt stress. MMW treatment improved seed germination and root growth under saline conditions and was associated with changes in the abundance of proteins involved in antioxidant defense, protein quality control, stress-related signaling, and carbohydrate metabolism. These findings identify candidate proteins and biological pathways that may contribute to MMW-mediated salt tolerance and provide a basis for a working model of the molecular responses induced by MMW treatment. However, because this study was conducted using a single rice cultivar under short-term salt stress conditions, the broader applicability of these findings remains to be validated. Further studies involving multiple cultivars, different stress conditions, long-term growth assessments, yield evaluations, and field trials will be necessary to clarify the practical value of MMW seed priming for improving crop performance in saline environments.

## Data Availability

MS data, RAW data, peak lists, and result files of proteomic analysis were deposited in the ProteomeXchange Consortium [68] via the jPOST [69] partner repository under data-set identifiers PXD056673.

## Supplementary Materials

The following supporting information can be downloaded at https://www.mdpi.com/article/10.3390/biology15211921/s1, Table S1. The list of primers used for gene expression analysis in this study, Table S2. The list of changed proteins in rice root under salt treatment compared with nontreatment of unirradiated seed, Table S3. The list of changed proteins in rice root under salt treatment compared with nontreatment of MMW irradiated seed, Figure S1. Coomassie-brilliant blue staining pattern for immunoblot, which used in Figure 5 and Figure 6, Figure S2. Blots of the entire membrane with anti-SOD antibody, which were used in Figure 5A, Figure S3. Blots of the entire membrane with anti-APX antibody, which were used in Figure 5B, Figure S4. Blots of the entire membrane with anti-PRX antibody, which were used in Figure 5C, Figure S5. Blots of the entire membrane with anti-GR antibody, which were used in Figure 5D, Figure S6. Blots of the entire membrane with anti-calreticulin antibody, which were used in Figure 6A, Figure S7. Blots of the entire membrane with anti-calnexin antibody, which were used in Figure 6B, Figure S8. Blots of the entire membrane with anti-calcium-dependent protein kinase antibody, which were used in Figure 6C, Figure S9. Blots of the entire membrane with anti-heat-shock protein 70 antibody, which were used in Figure 6D.

## Abbreviations

The following abbreviations are used in this manuscript:

APX	Ascorbate peroxidases
GR	Glutathione reductase
LC	Liquid chromatography
MMW	Millimeter wave
MS	Mass spectrometry
PCR	Polymerase-chain reaction
PRX	Peroxiredoxin
PVDF	Polyvinylidene difluoride
ROS	Reactive-oxygen species
SOD	Superoxide dismutase
AsA	Ascorbic acid
GSH	Glutathione
