# Supplementary material for: Millimeter-Wave Seed Priming Alters the Rice Root Proteomic Response to Salt Stress
Source: Biology (Basel). 2026 Oct 23;15(21):1921. doi: 10.3390/biology15211921 (PMC13761943; doi:10.3390/biology15211921)
Supplement: Supplementary file 1 [file biology-15-01921-s001.zip › Figures S1-S9.pdf]

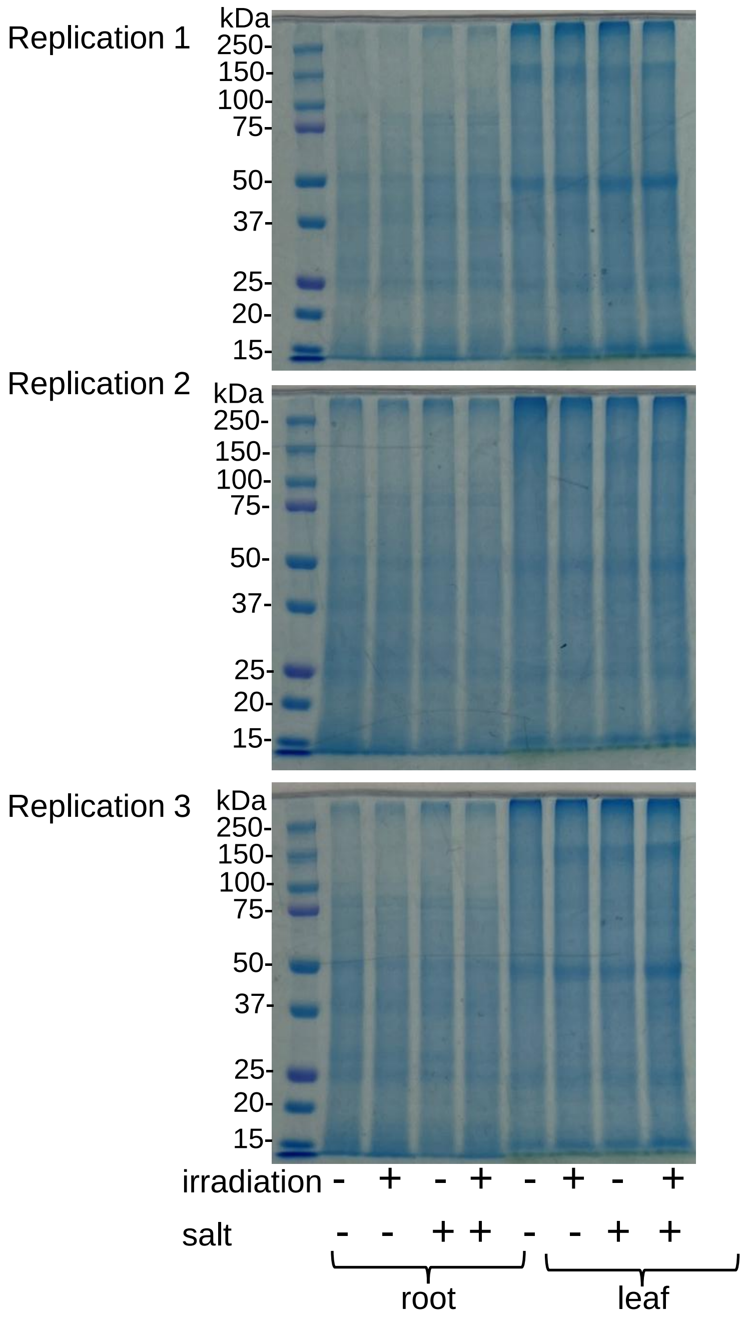


Figure S1. Coomassie-brilliant blue staining pattern for immunoblot, which used in Figures 5 and 6.

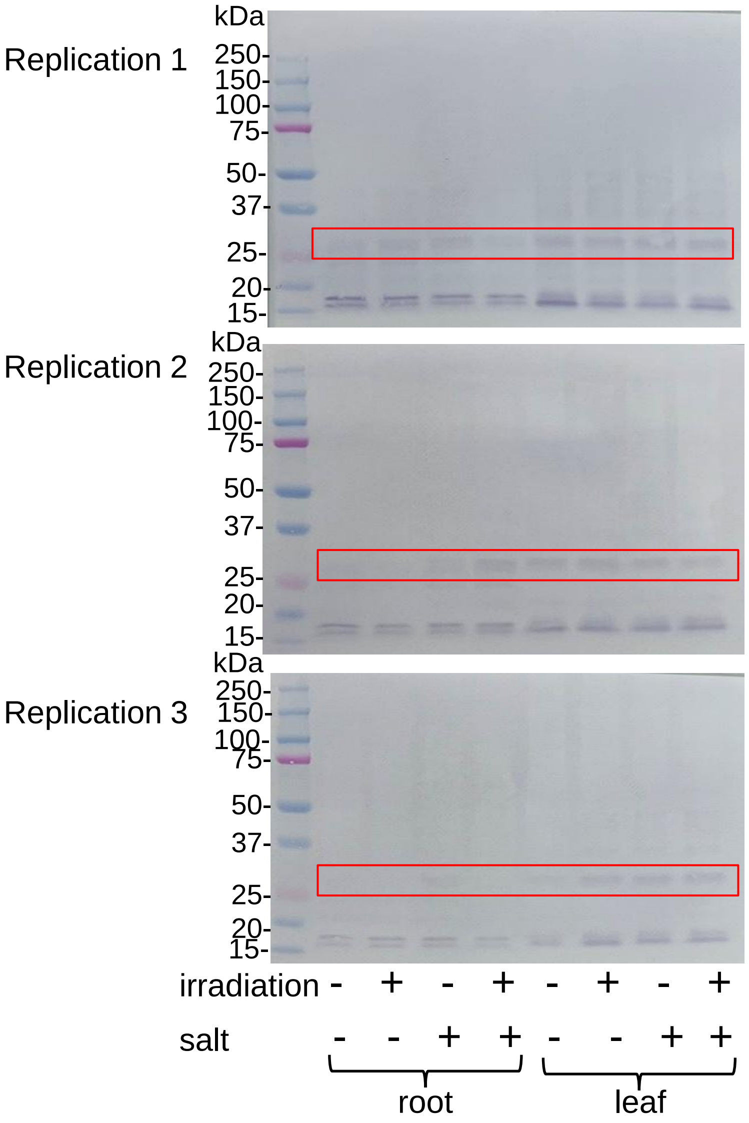


Figure S2. Blots of the entire membrane with anti-SOD antibody, which were used in Figure 5A.

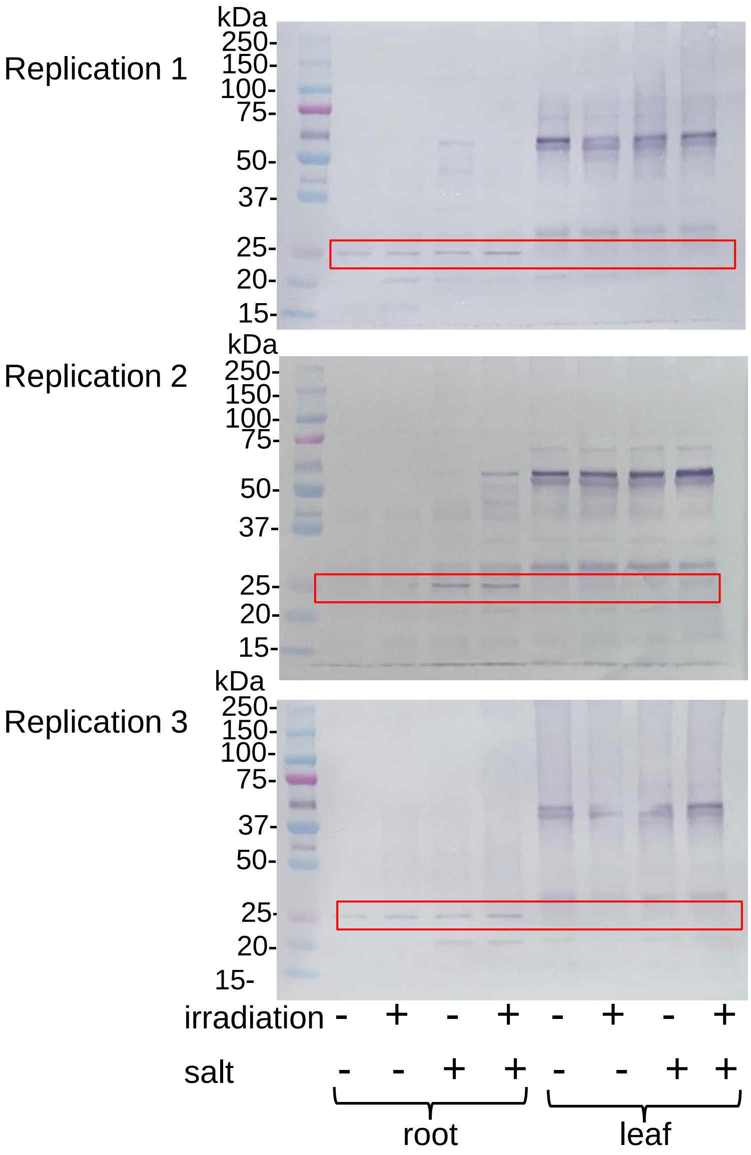


Figure S3. Blots of the entire membrane with anti-APX antibody, which were used in Figure 5B.

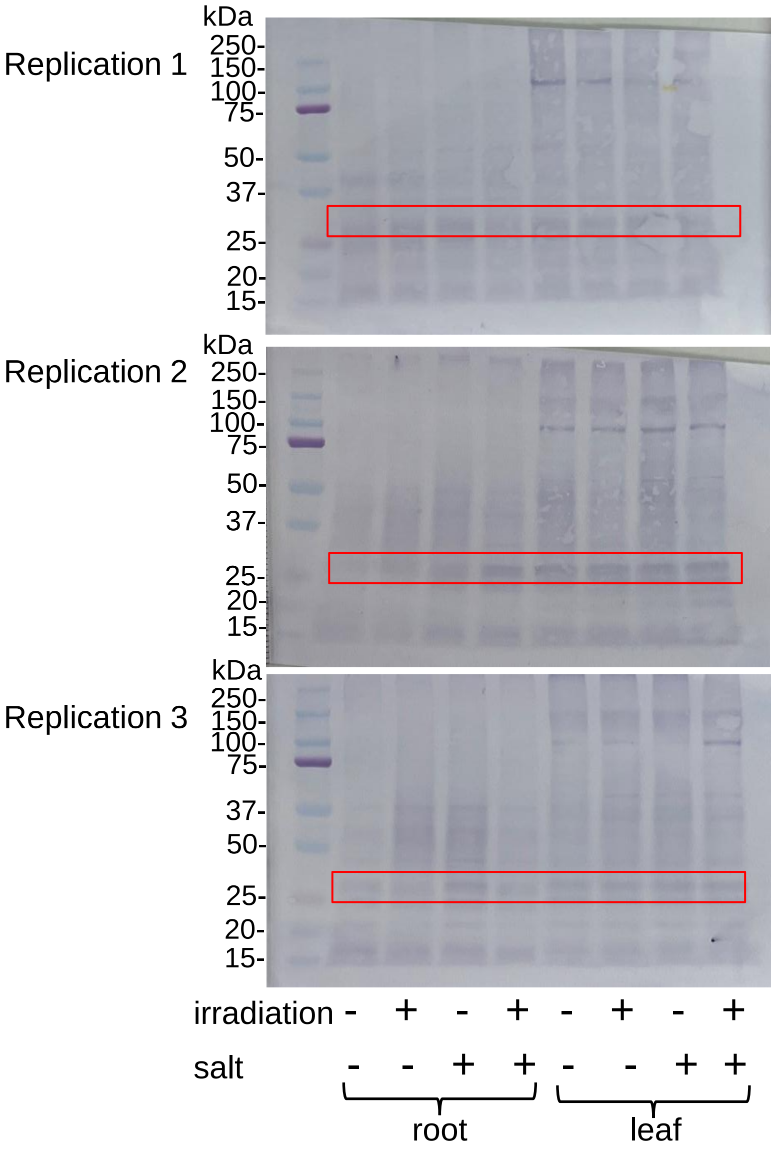


Figure S4. Blots of the entire membrane with anti-PRX antibody, which were used in Figure 5C.

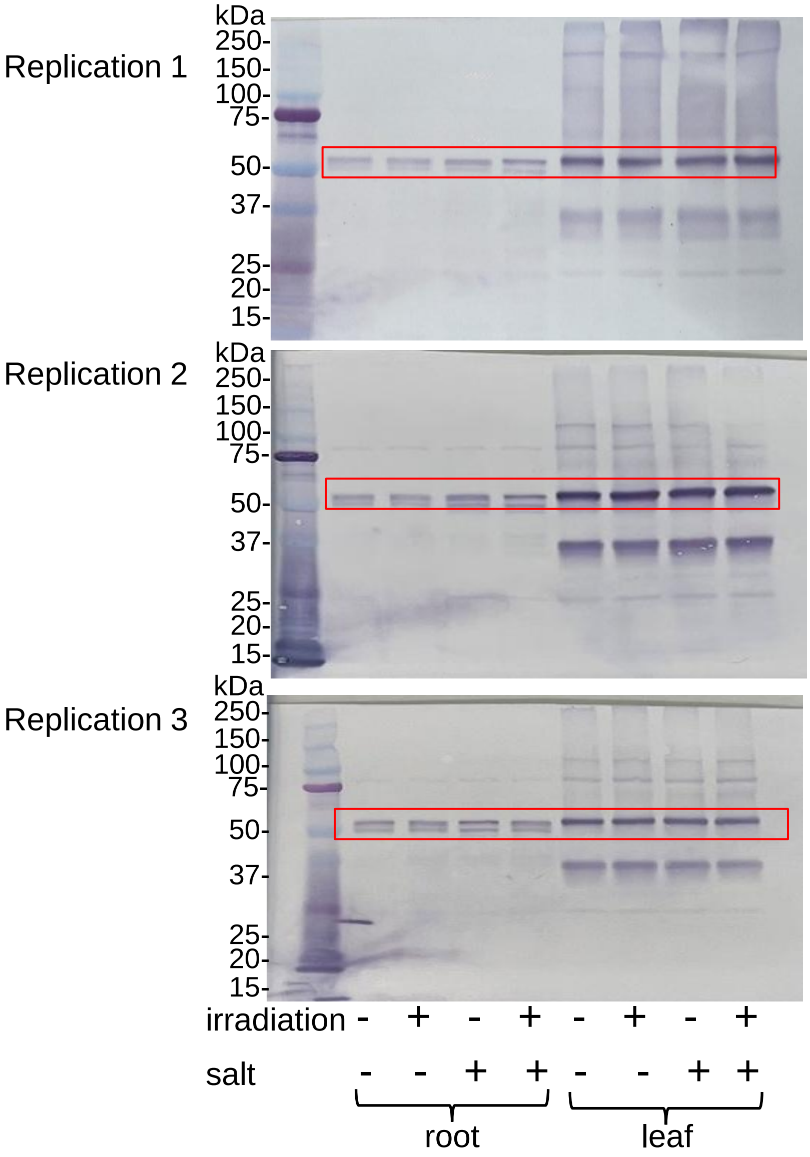


Figure S5. Blots of the entire membrane with anti-GR antibody, which were used in Figure 5D.

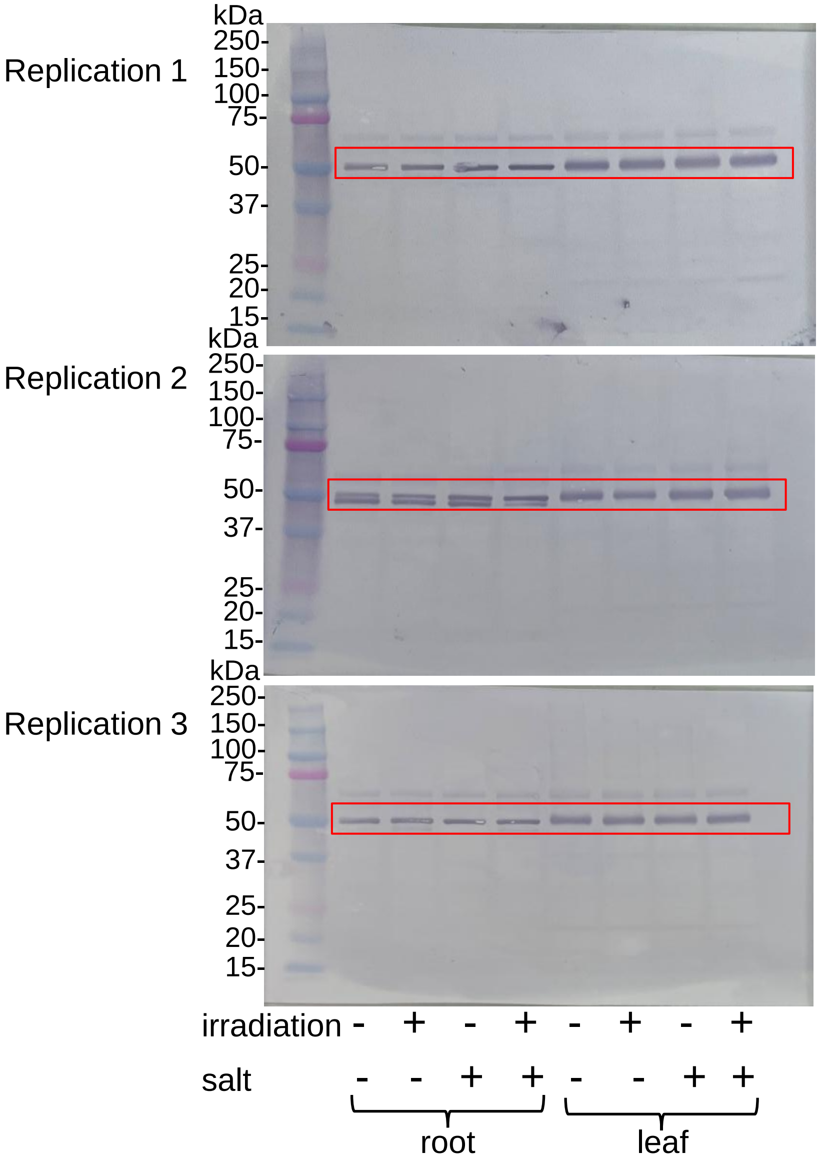


Figure S6. Blots of the entire membrane with anti-calreticulin antibody, which were used in Figure 6A.

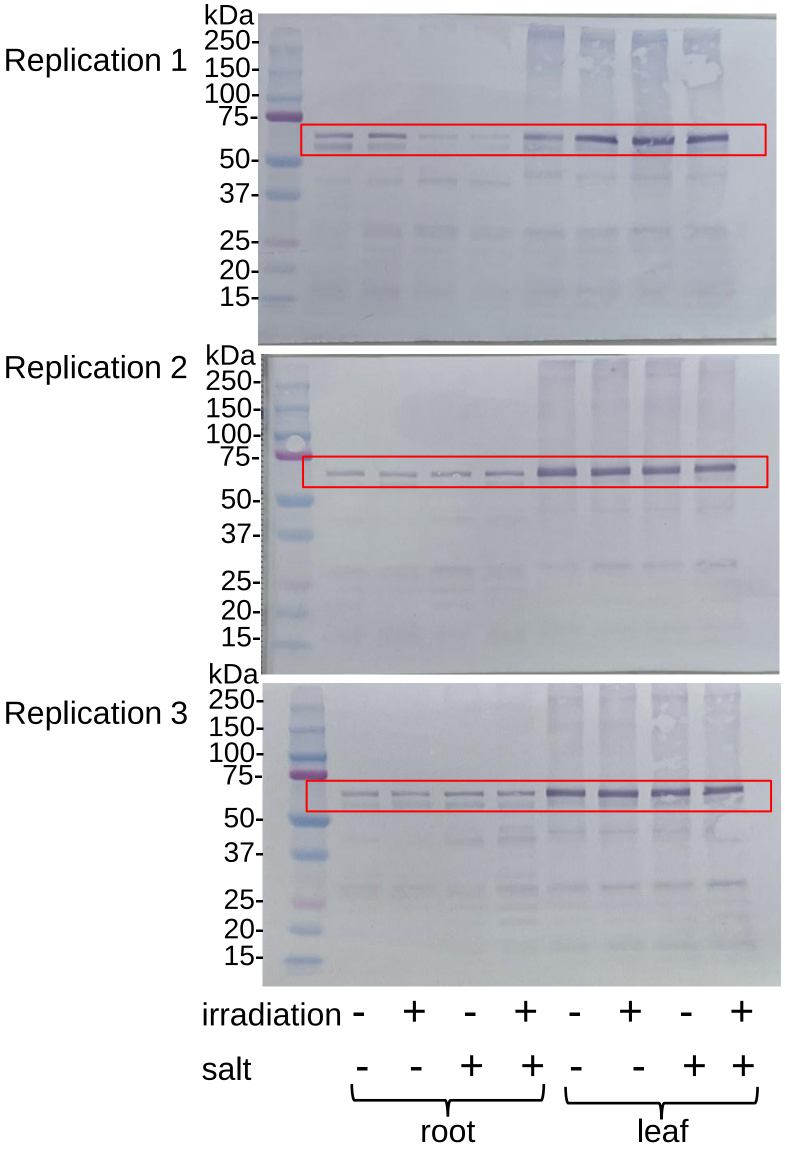


Figure S7. Blots of the entire membrane with anti-calnexin antibody, which were used in Figure 6B.

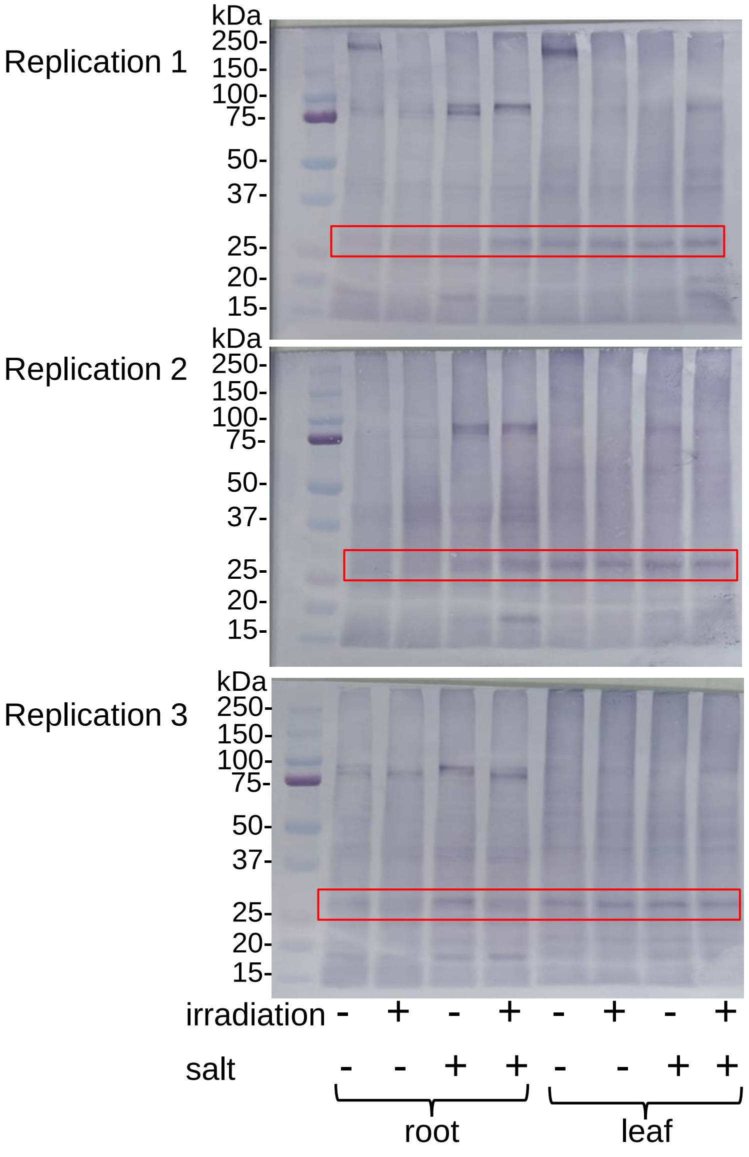


Figure S8. Blots of the entire membrane with anti-calcium-dependent protein kinase antibody, which were used in Figure 6C.

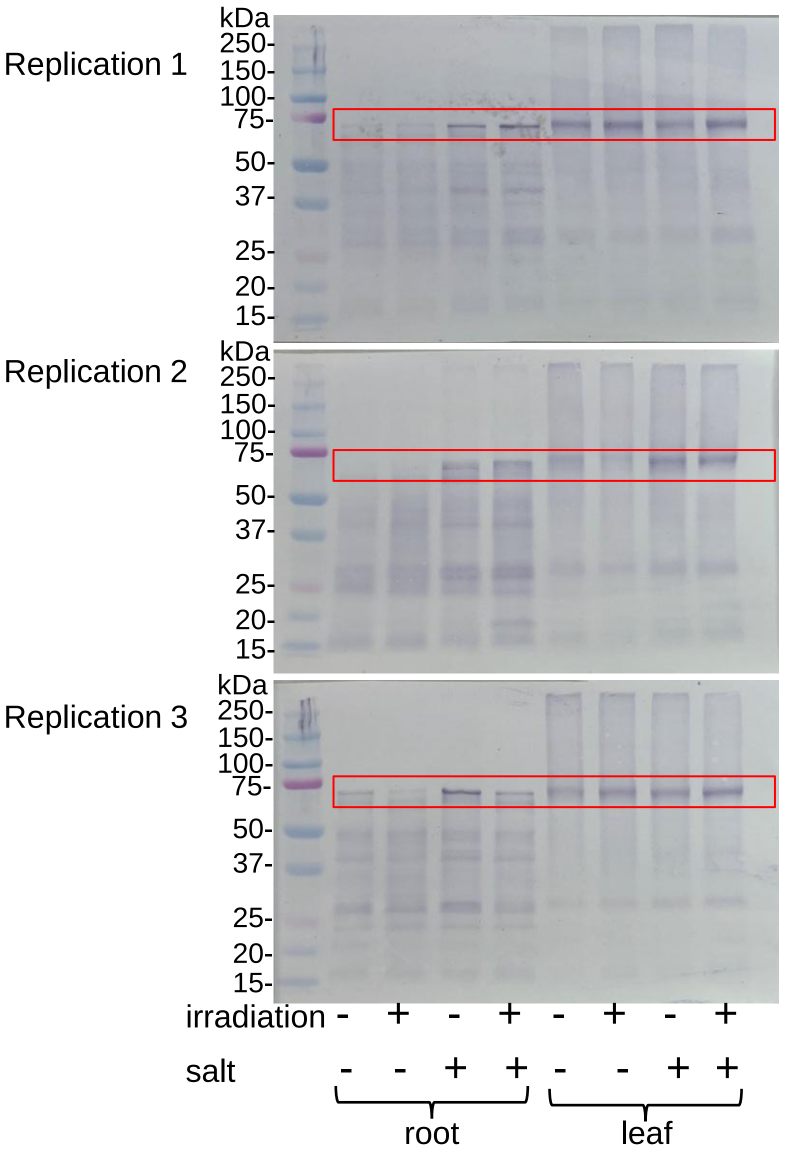


Figure S9. Blots of the entire membrane with anti-heat-shock protein 70 antibody, which were used in Figure 7D.
